# Behaviour of Pre-Cracked Self-Healing Cementitious Materials under Static and Cyclic Loading

**DOI:** 10.3390/ma13051149

**Published:** 2020-03-05

**Authors:** Giovanni Anglani, Jean-Marc Tulliani, Paola Antonaci

**Affiliations:** 1 Department of Structural, Geotechnical and Building Engineering (DISEG), Politecnico di Torino, Corso Duca degli Abruzzi 24, 10129 Torino, Italy; giovanni.anglani@polito.it (G.A.); paola.antonaci@polito.it (P.A.); 2 INSTM Research Unit PoliTO-LINCE Laboratory, Department of Applied Science and Technology (DISAT), Politecnico di Torino, Corso Duca degli Abruzzi 24, 10129 Torino, Italy

**Keywords:** self-healing, cementitious capsules, polyurethane, mechanical recovery, static loading, cyclic loading

## Abstract

Capsule-based self-healing is increasingly being targeted as an effective way to improve the durability and sustainability of concrete infrastructures through the extension of their service life. Assessing the mechanical and durability behaviour of self-healing materials after damage and subsequent autonomous repair is essential to validate their possible use in real structures. In this study, self-healing mortars containing cementitious tubular capsules with a polyurethanic repairing agent were experimentally investigated. Their mechanical behaviour under both static and cyclic loading was analysed as a function of some factors related to the capsules themselves (production method, waterproof coating configuration, volume of repairing agent stored) or to the specimens (number, size and distribution of the capsules in the specimen). Their mechanical performances were quantified in terms of recovery of load-bearing capacity under static conditions and number of cycles to failure as a function of the peak force under cyclic conditions. Positive results were achieved, with a maximum load recovery index up to more than 40% and number of cycles to failure exceeding 10,000 in most cases, with peak force applied during cyclic loading at least corresponding to 70% of the estimated load-bearing capacity of the healed samples.

## 1. Introduction

It is well-known that all cementitious materials tend to have defects, resulting from incorrect mix design or execution, shrinkage and other causes. Their low tensile strength, in combination with the presence of such defects, makes them prone to cracking. Because of the possible concurrent or subsequent action of cyclic loading, micro-cracks can propagate and coalesce into macro-cracks, ultimately leading to a reduction of durability or even collapse [1]. This is especially true for the infrastructure sector, where several bridges and viaducts are at risk due to the severe conditions caused by the increasing demand of traffic volumes [2,3,4].

Capsule-based self-healing has been proposed as a promising strategy to improve the durability and resilience of reinforced concrete infrastructures [5,6,7,8,9,10,11,12]. In particular, polyurethane (PU) precursors have been extensively investigated as possible core agents because of their ability to polymerise rapidly in the presence of humidity and hence to restore the material properties faster than other healing agents [13,14,15]. Their ability to restore good water impermeability performances has been widely established [9,16,17,18,19,20,21,22,23]. In addition, their capability to provide also a mechanical recovery has been highlighted in a few studies, mostly under static conditions [20,22,23,24]. Also, the effectiveness of different self-healing techniques under repeated cracking and healing cycles has been analysed in several studies [23,25,26,27,28,29,30]. However, as far as cyclic loading and fatigue performances are concerned, the mechanical behaviour of these materials has not been fully reported in the scientific literature, apart from few studies regarding self-healing systems under cyclic flexural loading [31] or under cyclic compressive loading [32]. Yet, the response to cyclic loading is particularly relevant from the mechanical point of view especially for the infrastructure sector, hence the importance of improving the behaviour of the materials commonly used in the sector, for example by adding different types of fibres [33,34,35] that can increase their performances in the event of cyclic loads [36,37,38]. In fact, the main sources of stress on infrastructures can be ascribed to dynamic actions, almost cyclically repeated over short periods of time or occurring with increasing intensity over longer periods of time. Moreover, cyclic loadings can cause additional detrimental effects on self-healing cementitious materials. Specifically, the cyclic actions lead to the continuous opening and closing of healed and unhealed cracks. In the case of healed cracks, the composite materials formed by the cementitious matrix and the repairing agent is subjected to strains that could cause not only damages in the matrix or in the repairing material, but also debonding between these two different materials. For this reason, it is of great importance to fill this knowledge gap by deepening the study of the behaviour of self-healing cementitious materials with respect to cyclic loading and fatigue. This is an issue of fundamental importance for understanding and characterising the behaviour of these materials in real field conditions, and consequently allowing their use by ensuring the structural safety.

Based on the above considerations and taking some of our previous experimental works as a starting point, the objectives of this study were to confirm the effectiveness of a capsule-based self-healing system using cementitious capsules and a polyurethanic repairing agent, to investigate the mechanical behaviour of the proposed system after pre-cracking under both static and cyclic flexural loading and to evaluate the influence of some capsule parameters and specimen configurations on the mechanical behaviour.

In order to pursue these objectives, cement mortar prisms either with or without capsules were used. Cement mortar was selected as a prototypal cementitious material instead of concrete for several reasons. First, the polyurethane reaction does not depend on the mortar fraction and the aggregate-cement ratio, hence it is easier to evaluate its effect in a more homogenous matrix as the one offered by mortar. In addition to the fact that mortar is more homogenous than concrete, the main reason for its use is the possibility to do a fast and cost-effective screening of different self-healing techniques before upscaling for the use in concrete [39]. For this reason, mortar is widely used in the literature on self-healing cementitious materials and consequently using it allows comparability with other techniques and testing methods.

The mechanical behaviour was investigated via three-point bending test in order to test the system under indirect tensile stresses and for the sake of comparison with the existing literature, it has been, so far, the most commonly employed test to assess the healing capacity of cementitious composites through the recovery of mechanical properties [40] under static loading conditions. Furthermore, the force controlled three-point bending test conducted by using a sinusoidal loading made it possible to investigate the mechanical behaviour until rupture of the pre-cracked and subsequently healed system. This allowed to obtain useful insights regarding the seldom studied fatigue performance of the self-healing systems, which is an aspect of paramount importance to develop a reliable preventive repair system that can be used in field conditions.

## 2. Materials and Methods 

### 2.1. Capsules

Cementitious tubular capsules were produced in accordance with previous researches [22,41,42,43] using a polymer-modified cement paste. The powder compounds of the paste were:

- Ordinary Portland cement (CEM I 52.5 R, Buzzi Unicem S.p.A., Casale Monferrato, Italy);
- Calcium carbonate (CaCO_3_, Sinopia s.a.s., Torino, Italy);
- Metakaolin (halloysite from Applied Minerals Inc., New York City, NY, USA, calcined at 650 °C for 3 hours);
- Hydroxypropyl methylcellulose (HPMC, Sigma Aldrich, Milano, Italy).

The liquid compounds of the paste were:

- Demineralised water;
- Copolymer of ethyl acrylate (EA) and methylmetacrylate (MMA) (Primal B60A, Sinopia s.a.s., Torino, Italy);
- Polyethylene glycol (PEG, Sigma Aldrich, Italy).

First, all solid compounds were manually mixed, while all liquid compounds were separately mixed together with an overhead stirrer (RW 20, IKA, Staufen, Germany). Then, the mixed powder compounds were added progressively to the homogenously mixed liquid compounds.

The fresh polymer-modified cement paste was used to produce cementitious tubes according to two different manufacturing processes.

In the first manufacturing process, already used in previous works [22,41,42,43], the fresh cement paste was manually extruded. This allowed to obtain ridged tubes of variable length with a hollow ovoid cross section (external diameter of 10 mm and internal diameter of 7.5 mm). They were left for 7 days in a moist environment (with temperature T ≈ 20 °C and relative humidity (RH) >95%) and subsequently in air (T ≈ 20 °C, RH ≈ 60%) for complete curing over 28 days. Afterwards, they were further cut with a saw into smaller tubes measuring about 45 mm in length.

In the second manufacturing process, the fresh cement paste was rolled around an oiled bar with a circular cross section (diameter of 5 mm), until obtaining a smooth cementitious tube with a shell thickness of approximately 1.5 mm. The bar was removed only after setting and first hardening of the cement paste, i.e., after 24 hours storage in a moist environment with T ≈ 20 °C and RH > 95%. This allowed to keep a perfectly cylindrical shape of the tubes, unlike the extruded ones in which deformation of the cross section occurred during the first stages of curing because of their own weight. The total duration of curing in moist environment was of 7 days, after which curing was completed in air (T ≈ 20 °C, RH ≈ 60%) over 28 days. At the end of curing, the tubes were further cut with a saw into smaller portions measuring about 60 mm in length.

**Figure 1:**
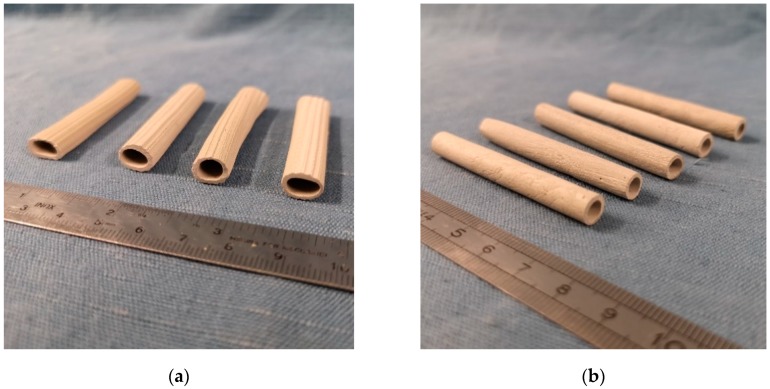
Cementitious tubular capsules produced by extrusion (**a**) and by rolling (**b**).

Figure 1 shows the cementitious tubes after saw cutting: on the left, the extruded tubes; on the right, the rolled ones.

The mix design of the cement paste was slightly modified with respect to previous works in order to accommodate both the extrusion process and the rolling procedure (see Table 1).

In order to avoid penetration of water through the capsule shell during concrete mixing and hydration, all the cementitious tubes produced were further coated. First, a two-component epoxy primer (Primer AQ, API SpA, Genova, Italy) was applied by complete immersion of the tubes in order to prepare the surface. Subsequently, a layer of two-component epoxy resin (Plastigel, API SpA, Italy) was applied to coat the capsule. The overall coating thickness was about 1 mm. The epoxy coating was applied to the internal surface of the tubes by injection in the case of the smaller capsules obtained by rolling (hence denoted as SI capsules). In the case of the larger capsules obtained by extrusion, the coating was applied either internally with the same procedure (LI capsules) or externally with a brush (LE capsules).

**Figure 2:**
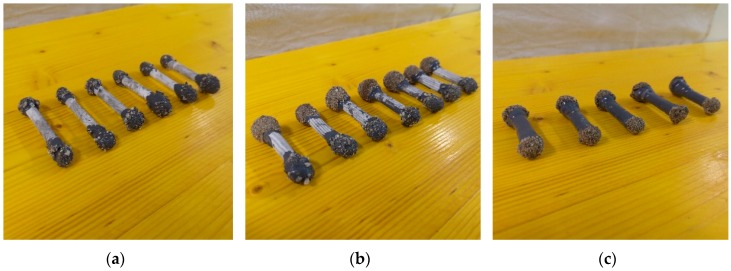
Cementitious capsules after coating, filling and sealing: (**a**) Small-diameter capsules with internal epoxy coating (SI capsules); (**b**) large-diameter capsules with internal epoxy coating (LI capsules); (**c**) large-diameter capsules with external epoxy coating (LE capsules).

After coating, one end of the tubes was sealed using an epoxy-based two-component thixotropic plaster (Stucco K, API SpA, Italy), further coated with Plastigel. At this point, the healing agent was injected with a syringe until complete filling. Finally, the second end of the tubes was sealed in the same way as the other and both ends were covered with sand in order to improve the bonding with the surrounding matrix. Pictures of the capsules thus obtained are reported in Figure 2.

The correct waterproofing and sealing of the capsules were issues of paramount importance, since a highly moisture-reactive substance was chosen as a healing agent. Namely, a commercially available polyurethane (PU) precursor (CarboStop U, Minova CarboTech GmbH Branch Italy, Milano, Italy) was used. It is a single-component resin that consists of modified polyisocyanates with additives that rapidly cures by reaction with ambient water yielding a polyurethane/polyurea foam. Its expansion rate depends on the backpressure exerted by the propagation of the resin into the structure to be sealed: wide cracks result in a high foaming factor, while narrow cracks in a low expansion rate and higher strength. Its viscosity range at 25 °C is 270–1,000 mPAs. The product is commonly used in the construction sector for stopping water inflow, water ingress in cracks and sealing of tunnel construction or of drill holes.

Table 2 summarises the main characteristics of each capsule series.

### 2.2. Mortar Prisms

Mortar prisms, with a volume of 40 by 40 by 160 mm^3^, were produced according to standard UNI EN 196-1. Ordinary Portland cement (CEM I 42.5 N, Buzzi Unicem S.p.A., Italy), tap water and normalised sand (grain size between 0.08 and 2.00 mm) were used, with a water-to-cement ratio of 0.5 and a sand-to-cement ratio of 3.

**Figure 3:**
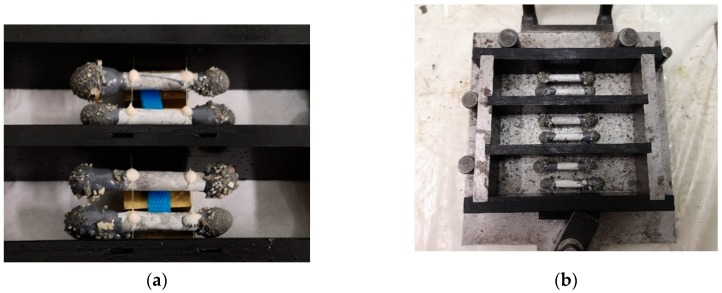
Mould used to produce the self-healing mortar prisms, before casting: (**a**) Bottom-up view of the capsules glued on the nylon threads with a methylmetacrylate (MMA) glue (X60, HBM, Darmstadt, Germany); (**b**) assembled and oiled mould with capsules in fixed position.

Reference specimens with no capsules (REF series) were produced along with self-healing specimens, in which the capsules filled with the PU precursor were embedded manually before casting, in fixed positions. Accurate control of the capsules position was achieved by gluing them on top of two thin nylon threads passing through the lateral faces of the mould, at a height of 10 mm from the bottom side of the specimen (see Figure 3).

Different series of self-healing specimens were produced: the CEM_SI series contained two small-diameter capsules with internal epoxy coating, the CEM_LI series contained one large-diameter capsule with internal epoxy coating, and finally the CEM_LE series contained one large-diameter capsule with external epoxy coating. CEM_SI and CEM_LI specimens contained the same volume of healing agent (~ 0.6 mL) but had different geometric configurations since the healing agent was distributed in two capsules or concentrated in one capsule, respectively. Conversely, CEM_LI and CEM_LE specimens had the same geometric configuration, with a single capsule horizontally centred in the specimen cross-section, but contained a different amount of healing agent (~ 0.6 mL and ~ 0.9 mL, respectively). For each series, 12 specimens were produced, for a total of 48 specimens.

**Figure 4:**
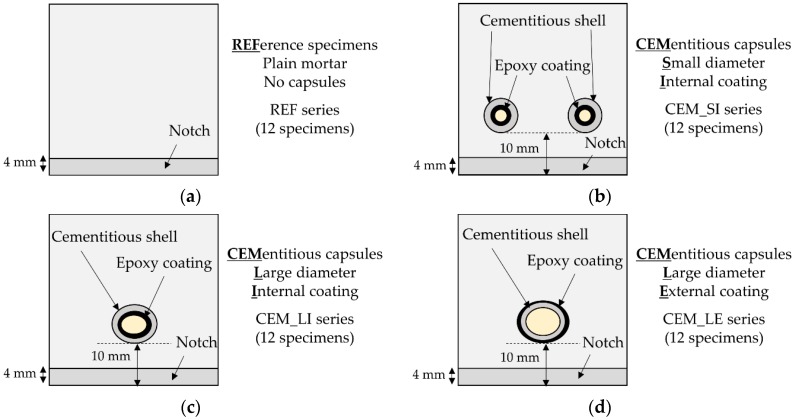
Schematic cross section of the specimens: (**a**) without capsules, REF series; (**b**) with two small-diameter capsules, CEM_SI series; (**c**) with one large-diameter capsule with internal epoxy coating, CEM_LI series; (**d**) with one-large diameter capsule with external epoxy coating, CEM_LE series.

After casting, the moulds were covered with plastic foils in order to maintain a humid environment (T ≈ 20 °C). The samples were demoulded after 24 hours from casting and cured for two weeks still wrapped in plastic foils (T ≈ 20 °C). Then, a U-shaped notch (width = 4 mm, height = 4 mm) was created by means of wet sawing. Figure 4 shows the schematic cross section of all the series.

### 2.3. Pre-Cracking

**Figure 5:**
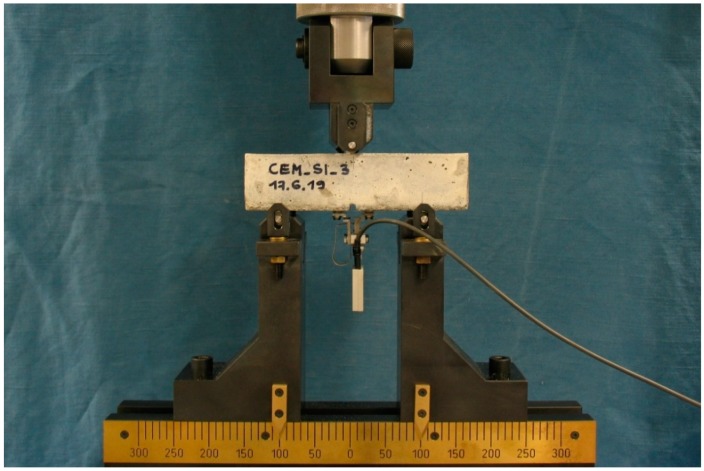
Setup of the pre-cracking procedure.

In order to evaluate the effectiveness of the autonomous self-healing system, a controlled localised crack was first induced in the specimens using a mechanical pre-cracking procedure. At an age of 14 days from casting, all the specimens were pre-cracked via a crack-width controlled three-point bending test with a span of 10 cm using a 25 kN closed-loop servo-controlled MTS hydraulic press (MTS 810, MTS Systems Corporation, Eden Prairie, MN, USA) equipped with a digital controller (FlexTest® 40, MTS Systems Corporation, USA). It was possible to control the crack mouth opening displacement (CMOD) by using a strain-gauge-based displacement transducer (DD1, range ± 2.5 mm, HBM, Germany) mounted on the bottom face of the specimen upon the notch. Figure 5 shows the setup of the pre-cracking procedure.

The test was conducted by imposing a CMOD rate of 1.5 µm/s. Upon reaching a maximum value of 800 µm, the specimens were unloaded at the same CMOD rate. The target value for the maximum CMOD was set at 800 µm in order to test the ability of the system to heal large cracks and in order to inhibit the influence of the autogenous healing, that could be significant for smaller cracks because of the young age of the specimens at pre-cracking. The CMOD and load were recorded throughout the execution of the test.

Immediately after pre-cracking, the specimens were stored in a curing cabinet (T = 20 °C, 60% RH) for one week and then in the lab, with no temperature and humidity control, for another week. Two supports with a span of 10 cm allowed to keep the specimens in the same geometric configuration during pre-cracking, with their bottom face pointing downward. The duration of the curing cabinet conditioning definitely exceeded the time required for the PU to react with air humidity and form a rigid foam (normally ranging from less than one hour up to a few hours, depending on ambient conditions). This ensured that the healing agent released from the capsules during pre-cracking was fully cured at the time of successive testing. 

### 2.4. Static Reloading

After 2 weeks from pre-cracking, the recovery of the mechanical properties was first evaluated by assessing the load-bearing capacity under static conditions.

Half of the specimens from each series were statically reloaded following the same procedure described in Section 2.3 for pre-cracking.

This allowed to evaluate the mechanical recovery through a load recovery index in accordance with common approaches reported in literature [20,22,40,41,42,44,45]. The load recovery index (LRI) was expressed as a function of the maximum load-bearing capacity of the specimens during pre-cracking (L_peak_) and reloading (L_reload_) and as a function of the residual load-bearing capacity at the end of pre-cracking (L_unload_). Its definition is reported in Equation (1):

LRI = (L_reload_ − L_unload_)/(L_peak_ − L_unload_). (1)

The static reloading allowed also to subsequently define the parameters to perform the cyclic reloading procedure (Section 2.5).

**Figure 6:**
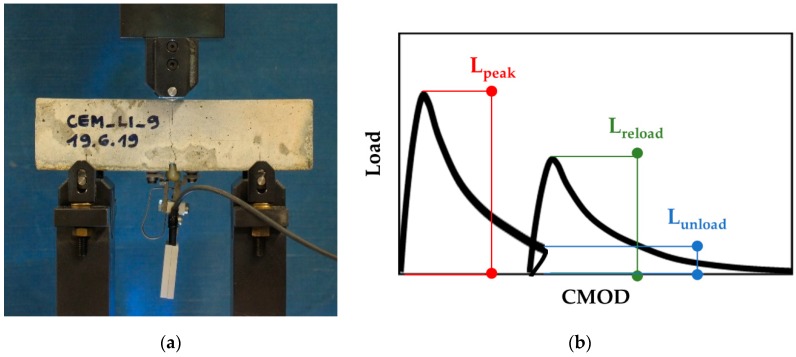
(**a**) Experimental setup for the static reloading procedure; (**b**) schematic load versus crack mouth opening displacement (CMOD) curves related to pre-cracking and reloading, with indication of maximum and residual load-bearing capacities.

Figure 6 reports the setup for the static reloading procedure and a graphical illustration of how the maximum and residual load-bearing capacities were calculated for the purposes of LRI determination.

### 2.5. Cyclic Reloading

After the static reloading of half of the pre-cracked specimens (Section 2.4), the mechanical behaviour under cyclic flexural loading was evaluated.

In order to investigate the behaviour in cyclic conditions, the remaining specimens were reloaded through a force-controlled three-point bending test with the same span used for the pre-cracking and static reloading (10 cm) and using the same closed-loop servo-controlled hydraulic press. In order to measure the damage evolution during the cyclic loading in terms of increment of crack opening displacement (COD), an inductive displacement transducer (WI, range 0–5 mm, HBM, Germany), connected to the digital controller through a measuring amplifier (SCOUT55, HBM, Germany), was mounted on the lateral surface of the tested specimen, just above the tip of the notch. It was not possible to mount the WI displacement transducer on the bottom face to measure the CMOD as in the pre-cracking and reloading setup because of the lack of space between the supports.

The force controlled three-point bending test was conducted by first reloading the specimens up to a maximum load value L_max_ with a load rate of 50 N/s. Upon reaching L_max_, a sinusoidal loading with a frequency of 4 Hz was applied, where the peaks were set to L_max_ and the valleys to a lower load value L_min_.

**Figure 7:**
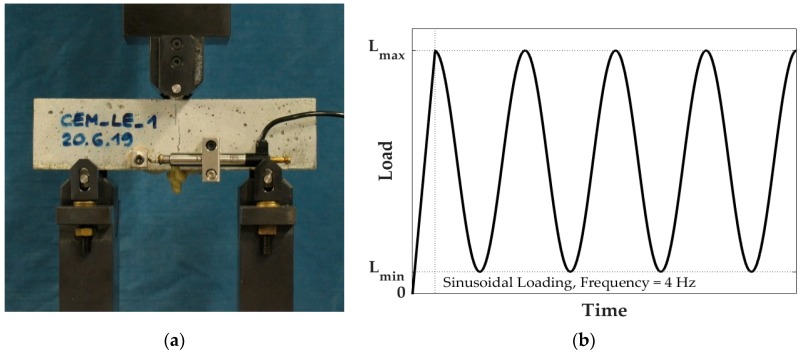
Cyclic reloading procedure: (**a**) experimental setup; (**b**) graphical illustration of the loading procedure for the cyclic reloading.

Figure 7 reports the experimental setup and a graphical illustration of the loading procedure used for the cyclic reloading.

The limits L_min_ and L_max_ of the cyclic reloading were defined as a fraction of the average load-bearing capacity L_reload_ obtained during static reloading for each series. Specifically, L_min_ and L_max_ were set according to Equation (2):

(2) $$\{\begin{array}{c} L_{\min}=0.1\cdot L_{reload}\\ L_{\max}=S_{}\cdot L_{reload} \end{array}$$

where the value of S was initially set to 0.7 for the first 10,000 cycles; if the specimen did not fail within the first 10,000 cycles, then the value of S was increased in steps of 0.05 for additional reloading series of 10,000 cycles each, until eventual failure of all the specimens. 

Such settings were adopted in order to impose a high stress, equal to or higher than 70% of the expected strength of the healed material. Indeed, after release and curing of the PU in the crack, the material properties changed in correspondence of the most stressed cross-section with respect to their initial values. They depended not only on the mechanical characteristics of the hardened PU, but also on its crack filling ratio and interaction with the surrounding cementitious matrix. The flexural strength of the healed material could only be estimated experimentally based on the load-bearing capacity L_reload_ obtained during static reloading. Testing the specimens cyclically with a cyclic peak value close to L_reload_ was meant to check their performances under severe conditions, in such a way to provide a first feedback on how this type of self-healing materials could behave during their remaining service life after they have produced their autonomous repair action. The number of cycles sustained until failure as a function of the maximum force applied during cyclic reloading was analysed as a representative parameter to describe their cyclic behaviour. 

## 3. Results and Discussion

### 3.1. Pre-Cracking

All specimens from each series (REF, CEM_SI, CEM_LI and CEM_LE), namely 12 specimens per series for a total of 48 specimens, were pre-cracked at an age of 14 days via a controlled three-point-bending test as described in Section 2.4.

**Figure 8:**
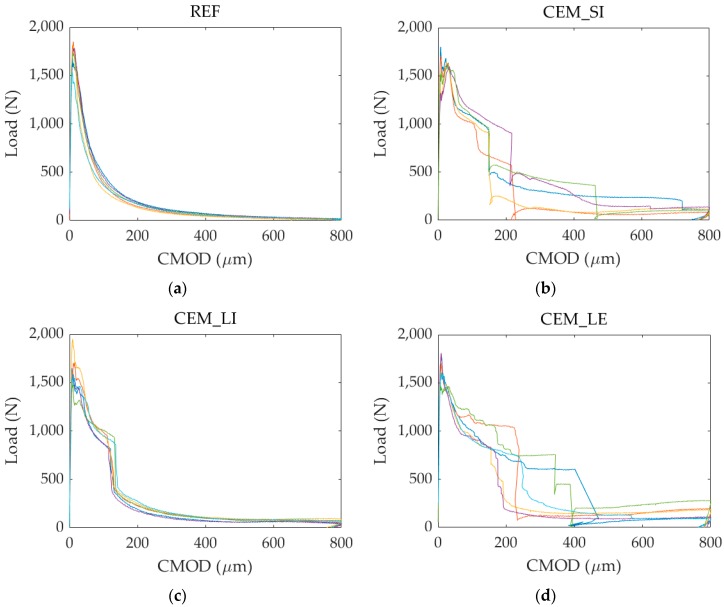
Load versus CMOD curves due to pre-cracking: (**a**) REF series; (**b**) CEM_SI series; (**c**) CEM_LI series; (**d**) CEM_LE series. The specimens are distinguished by different colour lines.

Figure 8 shows some of the load versus CMOD curves recorded during the tests for the different series. For the sake of clarity, only the curves related to the specimens that were later subjected to static reloading (see Section 3.2) are displayed.

During the controlled crack formation, first the load increased until reaching the peak value with a very low increase in terms of displacement because of the high stiffness of the intact material. After reaching the peak load, crack formation and subsequent propagation occurred, with progressive load decrease as a function of increasing CMOD due to the material post-peak softening behaviour. The specimens were unloaded upon reaching a CMOD of 800 µm, with a consequent small CMOD decrease due to recovery of the elastic deformation. While for the series without capsules (REF series) the softening phase had a continuous and gradual advancement, the self-healing series showed sudden load drops during this phase. These drops are attributable to the breakage of the capsules upon crack creation, being these drops accompanied with an audible acoustic energy release. These results are confirmed in literature, where the same behaviour was shown during crack creation using glass [20,24,46] and cementitious [22] capsules.

In all the specimens of the CEM_LI series, the drop was highly repeatable at a CMOD around 135 µm. In the case of the CEM_SI series, that contained two capsules per specimen, there were usually double load drops due to non-simultaneous breakage of the capsules. The first drop was always detected for a CMOD smaller than 215 µm. The highest scattering in the CMOD value corresponding to the load drop was shown by the CEM_LE series, for which such CMOD value did not exceed 240 µm on average: the higher scattering could be attributed to a higher variability in the coating thickness due to its manual application over the ridged surface of the capsule.

It is worth recalling here that the capsules were positioned 10 mm above the bottom face of the specimens, while the CMOD measuring points were situated some millimetres below it because of the experimental setup adopted. Therefore, the actual crack opening at the capsule level in correspondence of the aforementioned load drops was surely lower than the corresponding CMOD value experimentally detected. This results in the possibility of using the cementitious capsules to trigger the healing also for cracks smaller than 135–240 µm. This range of crack size is particularly relevant from a durability point of view, as confirmed by the structural design codes, where the limit for the maximum acceptable crack width is fixed to no more than 400 µm, depending on the exposure conditions [42,47,48].

**Figure 9:**
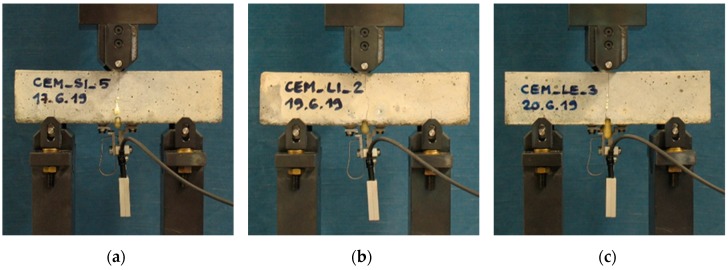
Leakage of polyurethane upon capsule breakage during pre-cracking: (**a**) Polyurethane (PU) visible both at the crack mouth inside the notch and on the lateral faces for a CEM_SI specimen; (**b**) PU visible at the crack mouth inside the notch for a CEM_LI specimen; (**c**) PU visible both at the crack mouth inside the notch and to a lesser extent on the lateral faces for a CEM_LE specimen.

A further evidence of the relationship between the load drops, the acoustic emission and the breakage of the capsules was that they were followed by leakage of the foaming polyurethane released by the capsules in the crack (see Figure 9).

The expansive foaming reaction is triggered by the capsule breakage, which makes it possible for the polyurethane precursor to get in contact with the humidity present in the air at the crack site and to start its polymerisation reaction. Such reaction induces gas formation and consequent expansion of the polyurethane precursor, that progressively passes from a viscous fluid state to a plastic foam and then hardened foam state. For equal conditions in terms of humidity, amount of reagent and relative contact surface, the expansion is governed by the crack width. It was possible to notice that in the case of the CEM_SI series containing two capsules, the PU leakage was most often visible both on the lateral faces and at the crack mouth, covering nearly the entire notch area. Despite that the amount of healing agent was the same, in the case of the CEM_LI series the PU leakage was mainly visible at the crack mouth, concentrated in the central part of the notch area in correspondence of the single capsule position. The CEM_LE series, containing 50% more healing agent with respect to the other two series, showed a behaviour in between, with the expansion zone mainly concentrated in the central part of the notch area in correspondence of the single capsule position, but with some visible leakage also from the lateral face. This releasing mechanism had consequences on the mechanical performance recovery which are dealt with in the following sections. 

Considering the peak load L_peak_, it should be pointed out that the presence of the cementitious capsules did not have a strong detrimental effect on the load-bearing capacity that the specimens could withstand. It is possible to assess the reduction of the load-bearing capacity L_reduction_ of the self-healing specimens with respect to the plain mortar specimens without capsules as:

L_reduction_ = (L_peak,REF_ − L_peak,i_)/L_peak,REF_, (3)

where L_peak,REF_ is the average load-bearing capacity L_peak_ calculated over the 12 specimens of the REF series and L_peak,i_ is the average load-bearing capacity L_peak_ calculated over the 12 specimens of each self-healing series. Respectively, the percentage reductions were equal to 8% for the CEM_SI series, 13% for the CEM_LI series and 6% for the CEM_LE series. This result showed that the flexural strength is not significantly influenced by the presence of the cementitious capsules, at least for the capsule proportion here used.

Table 3 summarises the results of the pre-cracking phase, with the average values and standard deviation of L_peak_ and L_unload_ per each series and the percentage load reduction L_reduction_ of each self-healing series with respect to the reference series.

### 3.2. Static Reloading

After the pre-cracking and the complete curing of the PU in a controlled environment (T = 20 °C, 60% RH), half of the specimens (6 per series, for a total of 24 specimens) were selected to be reloaded in static condition to evaluate the self-healing efficiency in terms of strength regain. The specimens were selected based on a visual inspection in order to always have another specimen with a comparable PU release to be tested later in cyclic condition (Section 3.3). 

**Figure 10:**
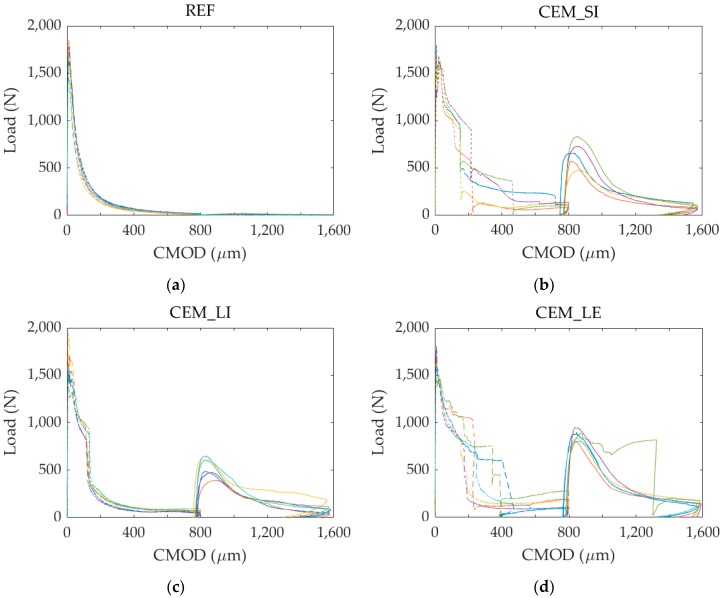
Load versus CMOD curves during the pre-cracking (dashed line) and the subsequent static reloading (continuous line) for the different series: (**a**) REF series; (**b**) CEM_SI series; (**c**) CEM_LI series; (**d**) CEM_LE series. The specimens are distinguished by different colour lines.

Figure 10 shows the load versus CMOD curves recorded during the pre-cracking and the static reloading for the different series. It is important to point out that the curves for the CEM_SI series are 5 because one specimen was accidentally broken during the positioning operations.

Upon reloading, the reference specimens without capsules could only reach at max the residual load-bearing capacity after pre-cracking (L_reload_ ≤ L_unload_) and then kept following the same softening branch of the pre-cracking phase. Conversely, the autonomously healed specimens showed a higher maximum load compared to the residual load-bearing capacity before healing (L_reload_ > L_unload_). The load versus CMOD curves were sufficiently repeatable and highlighted a slightly less brittle behaviour if compared to the intact specimen behaviour. The possibility to improve the ductility as a side effect of the self-healing through polyurethanic agents is a positive aspect in order to prevent brittle failure in the remaining life and to sustain higher deformation in service conditions.

The maximum load obtained during reloading was used to set the cyclic loading limits, as anticipated in Section 2.5, and to assess the load recovery in static condition according to the load recovery index definition reported in Equation (1). The reference specimens had negligible healing, in some case even negative performance. On the contrary, the self-healing specimens from all the series had satisfactory healing performances, with average LRI of about 36% for the CEM_SI series, 31% for the CEM_LI series and the best result of about 47% for the CEM_LE series (see Table 4 for detailed overview). This positive result is in good agreement with literature data concerning the use of cementitious capsules.

The manufacturing procedure of the cementitious capsule shells does not seem to influence the overall outcome of the system, since the load reduction during pre-cracking was negligible for all cases, as reported in Section 3.1. Also, the capsule breakage mechanism, with consequent polyurethane release, occurred in similar ways for either the capsules produced by extrusion or by rolling. 

Similarly, applying the epoxy coating to the internal or to the external surface of the tubular capsules does not appear to be a relevant factor of influence, considering that in both cases it allowed adequate waterproofing of the capsules and protection of the moisture-reactive healing agent, as confirmed by the abundant release of polyurethane during the pre-cracking stage for all the self-healing series. 

The volume proportion of healing agent seems to be correlated to the mechanical recovery under static condition, since better results were observed for the CEM_LE series, that contained 50% more polyurethane with respect to the other series. However, the increase in mechanical performance recovery is not directly proportional to the increase in healing agent volume, probably because in reality not all the polyurethane contained in the capsules reacted with ambient moisture upon crack formation, as it will be further discussed and exemplified in the following. Therefore, the increase in the mechanical performance recovery could be correlated to the internal diameter of the capsule more than to the overall internal volume. A larger internal diameter could be responsible for a better release in the cracked cross-section due to a more favourable ratio between capsule area and capsule perimeter, considering that the walls of the capsule inevitably cause a retaining action over the polyurethane.

For equal cumulative volume of healing agent, even if the capsule internal diameter is slightly smaller, the distribution of the capsules inside the specimen plays a more prominent role. Specifically, the use of more capsules increases the number of points from which the PU can be released and from which is allowed to expand, hence allowing a more even spreading of the PU over the crack surfaces, rather than a concentrated release in the central part of the section. This mechanism improves the overall crack filling and consequently should be reflected on the mechanical recovery during static reloading. This could explain the slightly superior performance of the CEM_SI series with respect to the CEM_LI series, although such conclusion needs to be further confirmed from a statistical point of view. 

To conclude the analysis of the static behaviour, one interesting case to be pointed out concerns one specimen of the series containing one large-diameter capsule with external epoxy coating, namely the specimen labelled as CEM_LE_9. Upon reloading, a hardening behaviour was detected after the peak value L_reload_, followed by a second load drop and a further polyurethane release. This event could be possibly ascribed to a self-protecting action of the polyurethane that sealed both the crack and the core of the capsule, protecting the unpolymerised precursor and allowing a second healing effect.

**Figure 11:**
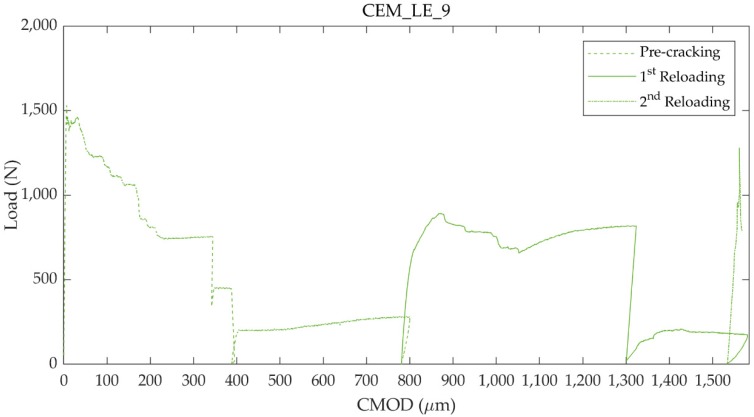
Load versus CMOD curves for the specimen CEM_LE_9 during the pre-cracking (CMOD up to 800 µm), the first static reloading in which there was a drop in load followed by a new PU release and the subsequent second static reloading after PU curing.

In order to analyse this second healing, after complete curing of the PU the specimen was further reloaded following the same procedure used for the pre-cracking. Figure 11 shows the load versus CMOD curves acquired for the pre-cracking, first reloading and second reloading procedures. It is to be pointed out that soon after reaching the new maximum load, the specimen was unloaded due to a malfunctioning in the control system of the testing machine.

Surprisingly, the new maximum load L_reload_ was higher than the one obtained in the previous reloading. It is possible to compare the two successive reloading in terms of LRI, comparing their maximum load with the peak load obtained during pre-cracking:

LRI_1_ = (L_reload,1_ − L_unload,p_)/(L_peak_ − L_unload,p_), (4)

LRI_2_ = (L_reload,2_ − L_unload,1_)/(L_peak_ − L_unload,1_), (5)

where L_peak_ is the load-bearing capacity obtained during pre-cracking, L_unload,p_ the residual bearing capacity after unloading during the pre-cracking, L_reload,1_ the maximum load carrying capacity obtained during the first static reloading, L_unload,1_ the residual bearing capacity after unloading during the first static reloading and L_reload,2_ the maximum load carrying capacity obtained during the second static reloading. The self-healing efficiency in terms of load carrying capacity regain increased in the second reloading to 82%, while in the first was equal to 50%.

Table 5 summarises these results regarding the static flexural behaviour of the specimen CEM_LE_9 during the pre-cracking and the successive two reloading stages.

### 3.3. Cyclic Reloading

After defining the limits of the cyclic flexural load as a function of the average load carrying capacity upon reloading L_reload_ per each series, the cyclic reloading itself was performed, in accordance with the procedure reported in Section 2.5. 

As expected, it was not possible to test the specimens without capsules under cyclic conditions. This was due to the fact that the selected pre-cracking procedure was used to simulate a severe condition of damage, resulting in a residual load carrying capacity after pre-cracking substantially nil for all the specimens. The plain mortar specimens without reinforcement of the REF series could not sustain any further load after pre-cracking because the contribution of the autogenous healing was insufficient to restore an adequate load carrying capacity, so that they failed immediately before the beginning of the actual cyclic test, during the positioning operations. On the contrary, the autonomous repair action provided by the polyurethane in the self-healing series allowed to perform the cyclic reloading in most cases. Specifically, all the specimens of the CEM_LI series were tested, (six in total) while in the case of the CEM_SI and CEM_LE series respectively three and two specimens failed in the initial part of the procedure, before reaching the prescribed cyclic peak load. It is worth reminding that the latter was defined as 70% of their estimated load-bearing capacity after healing. Clearly, the estimation based on the results of the static reloading test was not very accurate in these cases.

**Figure 12:**
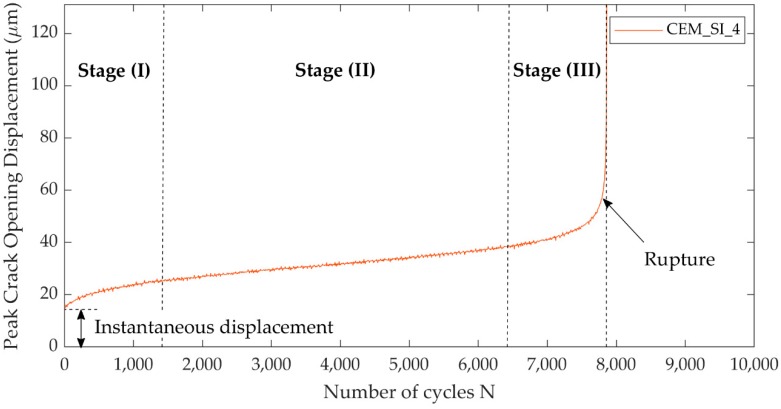
Three-stage fatigue evolution of the peak crack opening with increasing number of cycles.

For all the self-healing specimens that could undergo the cyclic reloading, a three-stage curve was detected. A typical example of it is reported in Figure 12, where the evolution of the crack opening at the peak of each cycle (i.e., at maximum load L_max_) is reported for the specimen CEM_SI_4.

This three-stage behaviour is typically observed in plain or fibre-reinforced concrete under compressive, tensile and flexural fatigue loading [36,49,50,51,52,53,54].

After an almost instantaneous crack opening displacement increase upon loading, there was a first rapid development denoted as flaw initiation, or Stage (I). In concrete, this stage involves the defects within the matrix; in the case of the self-healing system here studied, it most likely involved the defects at the interface between the PU and the crack faces and inside the PU foam, rather than the defects in the mortar matrix surrounding the crack, though it cannot be excluded that they also provided a contribution. It is worth noting that, in literature, tests conducted over pre-cracked fibre reinforced concrete under flexure lacked this stage, because the initial loading already caused the opening of the crack [55]. So, the experimental evidence of Stage (I) in cementitious mortar autonomously healed by PU is a further confirmation of the important role of the presence of the healing agent in restoring a behaviour similar to the one of an intact material.

The Stage (II) is a stable development stage that involves slow and progressive growth of damage, most likely concentrated in the bonding interface formed by the PU, and the progressive elongation and rupture of the foam cells. By comparison, in fibre-reinforced concrete this phase is mainly associated to the fibre/matrix bond deterioration [56,57] and fibre rupture [58].

Finally, the Stage (III) is the failure stage, in which damage develops unsteadily until fatigue failure occurs, with a complete failure of the bonding PU interface and detachment of the crack faces.

**Figure 13:**
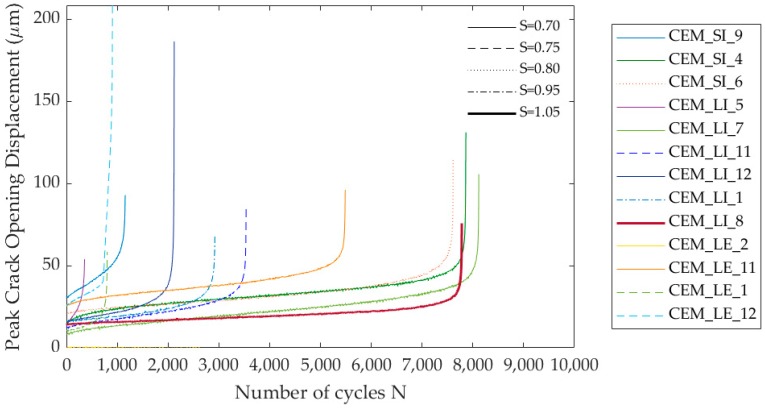
Peak crack opening versus number of cycles until complete failure, which occurred at different load level S among the specimens.

Figure 13 shows the curves that represent the relationship between the crack opening measured at the maximum load with the increasing of number of cycles N until complete failure, that occurred at different load level S, while Table 6 summarises the number of cycles sustained by each specimens at a given load level S.

The self-healing specimens were able to sustain a satisfactory amount of cycles before failure, both for the lower and the higher load levels. This result, compared to the performance of the reference specimens, strongly underlines the contribution of the autonomous self-healing system in restoring the mechanical properties of the damaged cementitious materials, especially in severe conditions of damage and also when cyclic loadings are involved, improving the resilience of the system during its remaining service life.

In terms of number of cycles and maximum load level, the best performance was expressed by the CEM_LI series. On the other hand, it is important to point out that this result deeply depends on the estimation of the load-bearing capacity after self-healing that was made based on the static reloading data (Section 3.2). 

Comparing the initial load-bearing capacity of the intact self-healing specimens and their residual load-bearing capacity after pre-cracking with the applied maximum load in cyclic condition, it is possible to see that the specimens sustained a maximum load in cyclic condition much higher than the residual bearing capacity after unloading in the pre-cracking phase (L_unload_): up to 15 times this residual capacity for the CEM_SI series, 10 times for the CEM_LI series and 5 times for the CEM_LE series (see Table 7). 

The comparison with the peak load of the intact specimens indicates that the system possesses a good recovery ability under cyclic conditions, being able to sustain cyclic loads up to 31% of the intact load-bearing capacity for the CEM_SI series, up to 36% for the CEM_LI series and up to 38% for the CEM_LE series (see Table 7). This is in good agreement with the performance regain observed under static conditions, where the values of the load recovery indices were approx. 36%, 31% and 47% respectively.

**Figure 14:**
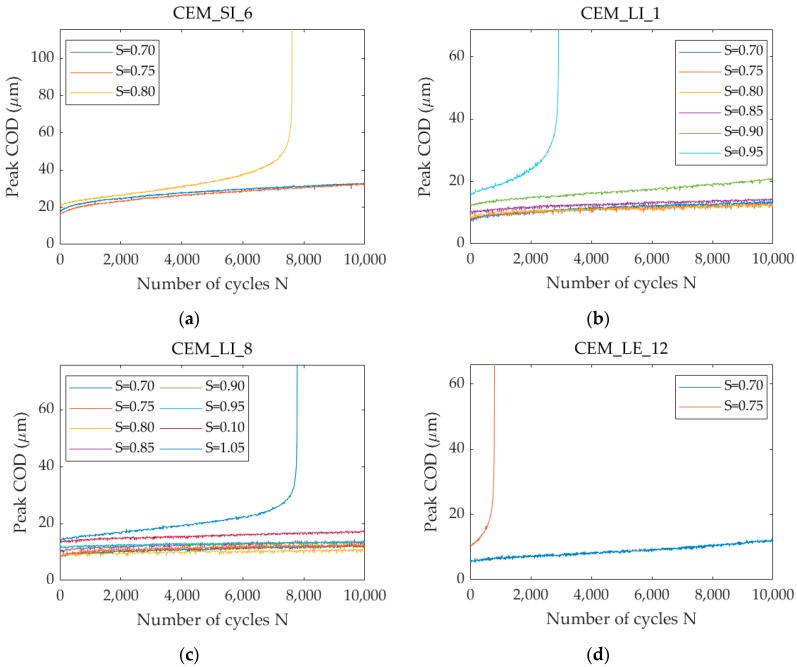
Evolution of the crack opening displacement at the maximum load with increasing number of cycles N with increasing load level S: (**a**) CEM_SI_6; (**b**) CEM_LI_1; (**c**) CEM_LI_8; (**d**) CEM_LE_12.

As a general tendency, looking at Table 6 it is possible to notice an overall decrease in the number of cycles to failure with increasing load levels. Figure 14 shows the relationship between the evolution of the crack opening at the maximum load with increasing number of cycles N, at each load level S at which they were tested before complete failure. In the figure, four of seven specimens that underwent the cyclic testing at increasing load levels are displayed, as a way of example.

Looking in detail at these curves, it is possible to notice that with increasing S an increase of the slope in the Stage (II) curves occurs and a progressive deviation from linearity as a function of the number of cycles is displayed. The latter is a typical feature of the Stage (III) and is evidenced for example in Figure 14b for S = 0.90, after about 7,000 cycles. This feature is directly correlated to a faster increase in the damage rate evolution. Further studies would be needed to better investigate this change of slope in correlation with the load level S, for the purpose of characterising the fatigue life of the self-healing cementitious materials, in comparison with ordinary and fibre reinforced concrete. Nevertheless, these results represent a step forward with respect to the limited literature concerning the fatigue performances of self-healing cementitious systems, which is an important knowledge gap that needs to be filled in order to ensure the reliability of these systems in real field conditions.

## 4. Conclusions

In this study, self-healing mortars were produced by incorporating cementitious tubular capsules filled with a polyurethane precursor. The capsules were manufactured according to two different procedures, by extrusion and by rolling of a polymer-modified cement paste, and were also characterised by different waterproofing coating configurations, size and distribution within the mortar specimens. Their self-healing effect was evaluated in terms of mechanical recovery under static flexural loading and number of cycles to failure under cyclic flexural loading. The results were not significantly affected by the capsule manufacturing procedure and by the sequence of application of the epoxy coating layers; conversely, the internal diameter of the capsules and their distribution within the specimen may have influenced the release of the polyurethane precursor upon pre-cracking and hence the final mechanical performance. In all cases, the mechanical regain was very satisfactory in comparison with the behaviour of reference plain mortars, both under static and cyclic loading. A maximum load recovery index in static condition of more than 40% and a maximum number of cycles to failure of more than 70,000 were observed, confirming the potential of the proposed capsule-based self-healing system for the purposes of improving the structures durability and mechanical performances.

## Figures and Tables

**Table 1:** Mix design of the polymer-modified cement paste.

Cement (wt %)	Water (wt %)	Water/Cement Ratio (-)	CaCO_3_ (wt %)	Metakaolin (wt %)	HPMC (wt %)	Primal B60A (wt %)	PEG (wt %)
46.2	12.8	0.28	21.3	0.3	0.7	17.0	1.7

**Table 2:** Main characteristics of the different capsule series.

		SI Capsule	LI Capsule	LE Capsule
Manufacturing process		Rolling	Extrusion	Extrusion
Surface of the tubular shell coated with epoxy		Internal	Internal	External
Average internal diameter of the tubular shell	(mm)	5	7.5	7.5
Average external diameter of the tubular shell	(mm)	8	10	10
Average length of the capsule	(mm)	60	45	45
Average thickness of the epoxy coating	(mm)	1	1	1
Average internal diameter after epoxy coating	(mm)	3	5.5	7.5
Injected volume of PU precursor	(mL)	~ 0.3	~ 0.6	~ 0.9

**Table 3:** Overview of the average L_peak_ and L_unload_ (with respective standard deviation) and L_reduction_ for each series (12 specimens per series) after the pre-cracking procedure.

Series	L_peak_	L_unload_	L_reduction_
	(N)	(N)	(%)
REF	1844 ± 140	14 ± 5	-
CEM_SI	1694 ± 86	88 ±2 4	8
CEM_LI	1611 ± 147	59 ± 15	13
CEM_LE	1737 ± 125	143 ± 58	6

**Table 4:** Overview of L_peak_ and L_unload_ (average values and standard deviations over 12 specimens per series), L_unload_ and LRI (average values and standard deviations over six specimens per series apart CEM_SI ^1^).

Series	L_peak_	L_unload_	L_reload_	LRI
	(N)	(N)	(N)	(%)
REF	1844 ± 140	14 ± 5	15 ± 9	0.1 ± 0.4
CEM_SI	1694 ± 86	88 ± 24	659 ± 137 ^1^	35.9 ± 17.4 ^1^
CEM_LI	1611 ± 147	59 ± 15	547 ± 105	30.8 ± 6.7
CEM_LE	1737 ± 125	143 ± 58	869 ± 57	46.5 ± 3.6

^1^ Estimated on five reloaded samples.

**Table 5:** Overview of the parameters characterizing the static flexural behaviour of the CEM_LE_9.

Series	No.	L_peak_	L_unload,p_	L_reload,1_	LRI_,1_	L_unload,1_	L_reload,2_	LRI_,2_
		(N)	(N)	(N)	(%)	(N)	(N)	(%)
CEM_LE	9	1531	258	896	50	165	1280	82

**Table 6:** Number of cycles sustained by the specimens at each load level S.

Load Level S		0.70	0.75	0.80	0.85	0.90	0.95	1.00	1.05
Series	No.	Number of Cycles N							
REF	all	0	-	-	-	-	-	-	-
CEM_SI	9	1177	-	-	-	-	-	-	-
	4	7881	-	-	-	-	-	-	-
	6	10,000	10,000	7,625	-	-	-	-	-
CEM_LI	5	381	-	-	-	-	-	-	-
	7	8,138	-	-	-	-	-	-	-
	11	10,000	3,557	-	-	-	-	-	-
	12	10,000	10,000	10,000	2,141	-	-	-	-
	1	10,000	10,000	10,000	10,000	10,000	2,940	-	-
	8	10,000	10,000	10,000	10,000	10,000	10,000	10,000	7,797
CEM_LE	2	2,732	-	-	-	-	-	-	-
	11	5,503	-	-	-	-	-	-	-
	1	10,000	816	-	-	-	-	-	-
	12	10,000	921	-	-	-	-	-	-

**Table 7:** Comparison between the load-bearing capacity of the intact self-healing specimens and their residual load-bearing capacity after pre-cracking with the applied maximum load in cyclic condition.

		Load Level S							
	Series	0.70	0.75	0.80	0.85	0.90	0.95	1.00	1.05
L_max_/L_unload_	CEM_SI	13	14	15	-	-	-	-	-
	CEM_LI	6.5	7	7.5	8	8.5	9	9.5	10
	CEM_LE	4.5	5	-	-	-	-	-	-
L_max_/L_peak_	CEM_SI	0.27	0.29	0.31	-	-	-	-	-
	CEM_LI	0.24	0.25	0.27	0.29	0.31	0.32	0.34	0.36
	CEM_LE	0.35	0.38	-	-	-	-	-	-
